# Towards a Stable Host–Parasite Relationship Between Honey Bees and *Varroa* Mites Through Innovative Beekeeping

**DOI:** 10.1111/1462-2920.70101

**Published:** 2025-05-01

**Authors:** Lioba Hilsmann, Lena Wolf, Markus Thamm, Sylvie Vandenabeele, Ricarda Scheiner

**Affiliations:** ^1^ Behavioral Physiology and Sociobiology Biocenter, Julius‐Maximilians‐Universität Würzburg Würzburg Germany

**Keywords:** evolution/evolutionary processes/gene transfer/mutation, microbe: virus interactions, microbiological‐based health strategies, pathogen ecology, viruses and their biology

## Abstract

*Varroa destructor* is a major factor in declining honey bee health worldwide. Conventional beekeeping involves multiple *Varroa* treatments, limiting bees' ability to adapt to the mite. To foster a stable host–parasite relationship, we tested an “innovative” beekeeping method with fewer *Varroa* treatments, focusing on its impact on honey bee health. We compared *Varroa* mite fall, immune responses, and seasonal dynamics of Deformed Wing Virus‐B (DWV‐B) in colonies managed under conventional and innovative practices. Viral loads of newly emerged honey bees and foragers were quantified three times during the season. *Varroa* mite fall was monitored and immune responses were assessed. In spring, bees managed with the innovative method had significantly lower haemocyte counts 48 h after emergence. DWV‐B loads did not differ between groups in spring but were higher in summer in bees managed with the innovative method. After summer treatment, DWV‐B loads and *Varroa* mite fall were similar between groups. Despite higher numbers in *Varroa* mite fall and DWV‐B loads in summer, the innovative method reduced both by fall, ensuring healthy winter bee production and colony survival. These findings suggest that reducing *Varroa* treatments can support a stable host–parasite relationship while minimising negative effects on honey bee health.

## Graphical abstract

An innovative *Varroa* treatment increases *Varroa* pressure and Deformed Wing Virus (DWV‐B) load in summer. The increased mite and virus levels are reduced with effective summer treatment so that finally there is no difference between both groups in terms of DWV‐B and mite load shortly before overwintering.

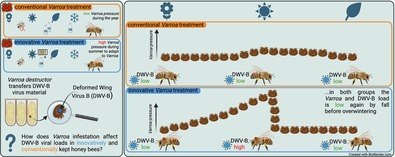

## 1 Introduction

The loss of honey bee colonies is a major threat to beekeepers around the world (Genersch et al. 2010). Especially in winter, beekeepers have to deal with severe colony losses, termed “winter losses”. In the United States, beekeepers lose approximately 50% of their colonies every year (Lamas and Evans 2024). In Germany, an average winter loss rate of between 6.6% and 16.7% was calculated for the last 17 years (Rosenkranz et al. 2021). Worldwide, winter losses ranging from 5.8% to 32.0% were observed in the winter of 2018 to 2019 (Gray et al. 2020). Honey bee colony losses have been intensively investigated in the past because of the massive impact of honey bees on apiculture and the importance of honey bees in economy and ecology. Reasons for the death of entire honey bee colonies can be their weakness, for example due to poisoning by pesticides or a high virus loads in late fall. In addition, old queens can lead to colony collapse (Genersch et al. 2010; Hristov et al. 2020).

The main cause for colony losses is generally attributed to an infestation with *Varroa destructor* (Anderson and Trueman), an ectoparasitic mite (Hristov et al. 2020). The mite originally stems from the eastern honey bee *Apis cerana* (Rosenkranz et al. 2010; Traynor et al. 2020). In the 1950s, *Varroa* spread from the Asian continent and its original host to the rest of the world (Traynor et al. 2020; Oldroyd 1999). Australia has long been the only continent without *Varroa* mites. However, the *Varroa* mites were detected in numerous properties in New South Wales in 2022 (Hetherington 2023). Despite all efforts, the spread of the mite in Australia could not be stopped, which means that *Varroa* has now spread to all continents. This underlines the urgent need for allowing the honey bee to adapt to the mite, because there are hardly any *Varroa*‐free populations of honey bees available world‐wide.

An infestation has serious consequences for *Apis mellifera* . On an individual level, *Varroa* parasitism can lead to a body weight loss of 6.3%–25% in worker bees (de Jong et al. 1982). Honey bees infested during the pupal stage show differences in physiological traits compared to uninfested honey bees (Amdam et al. 2004; Zanni et al. 2018). Non‐associated learning and navigation skills are also negatively affected by *Varroa* infestation (Rosenkranz et al. 2010). *Varroa* parasitism can also negatively affect the immune response of honey bees by reducing the total amount of haemocytes (Yang and Cox‐Foster 2007; Belaϊd and Doumandji 2010; Koleoglu et al. 2018). These honey bee blood cells play a key role in the cellular and humoral immune system. They are required for defence mechanisms such as wound healing, melanization, and encapsulation (Negri et al. 2016).

Most importantly, *Varroa* acts as a vector for honey bee diseases (Traynor et al. 2020; Wilfert et al. 2016; Bowen‐Walker et al. 1999). *Varroa* can transmit DWV and Acute bee paralysis virus (ABPV) to honey bees (de Miranda and Genersch 2010; Martin and Brettell 2019; Traynor et al. 2020; Noël et al. 2020). DWV belongs to the *Iflaviridae* family and was first detected in 1982 (Bailey and Ball 1991). The virus contains one copy of single stranded positive sensed RNA (Martin and Brettell 2019). There are four master variants of DWV, that is, DWV‐A, DWV‐B, DWV‐C and DWV‐D (Paxton et al. 2022; de Miranda et al. 2022). Since DWV‐C and DWV‐D are not the prevailing genotypes, most studies have focused on the other two master variants. DWV‐A, the first master variant discovered, is highly associated with colony collapses (Schroeder and Martin 2012). DWV‐B was first described in 2004 as *Varroa destructor* virus 1 (VDV‐1) (Kevill et al. 2019). VDV‐1 was later renamed DWV‐B, as the two variants DWV‐A and DWV‐B show a convergence of 84% of genome identity (Ongus et al. 2004). DWV‐B is now observed in several countries around the world, for example, in Germany (Natsopoulou et al. 2017), France (Wilfert et al. 2016), the United Kingdom (McMahon et al. 2016), Israel (Zioni et al. 2011) and the United States (USA) (Ryabov et al. 2017). Interestingly, there has been a shift in the prevalence of DWV‐A and DWV‐B in temperate regions in the past (Kevill et al. 2021). While the incidence of DWV‐A decreased, the prevalence of DWV‐B increased (Kevill et al. 2021; Doublet et al. 2024). It is not yet known whether DWV‐B is the more dangerous variant for honey bee colonies, as there are differences in transmission efficiency and virulence (Paxton et al. 2022). Intriguingly, the replication of DWV‐B virus does not only take place in the honey bee but also in the mite itself (Gisder and Genersch 2021; Ongus et al. 2004). In German honey bee colonies, DWV‐B is the most prevalent master variant (Natsopoulou et al. 2017). Natsopoulou et al. (2017) suggest that a lack of brood during winter in temperate zones, combined with the high virulence of DWV‐B, can lead to the premature death of honey bees and entire colonies, even when *Varroa* mite infestations are low. DWV‐B can therefore also lead to the death of honey bee colonies, even if beekeepers treat the hives to reduce the *Varroa* infestation.

To avoid colony losses, beekeepers treat their colonies against *Varroa* infestation. Commonly used beekeeping practice lowers the infestation rate throughout the entire year. Drone brood is regularly removed during the mating season (April–June) to reduce *Varroa* load, because the mites prefer to infest drone brood. In addition, the mite population can be further reduced by treating colonies with formic acid in late summer (Brodschneider et al. 2022). Treatment with formic acid is a well‐established method in beekeeping practice with an efficiency of between 75% and 86% when applied in classical doses (Bachert et al. 2022). In winter, treatment with oxalic acid is performed when the hives are naturally brood free. Oxalic acid is also well‐established as a treatment against *Varroa* infestation (Brodschneider et al. 2022; Charriére and Imdorf 2002). This combination of treatments, termed “**conventional beekeeping**”, leads to a constantly low mite load and therefore low pressure on honey bee colonies. However, the specific management strategy and choice of treatment vary widely between regions worldwide and are adapted to environmental conditions (Brodschneider et al. 2022; Jack and Ellis 2021).

The permanent reduction of *Varroa* mites in managed honey bee colonies likely prevents a stable host–parasite relationship. A controlled mite pressure throughout the season would be required to allow for a possible adaptation of the bees.

In an **“innovative beekeeping”** concept, high mite pressure is to foster a stable host–parasite relationship in the long run and to allow for the development of resistance mechanisms against the mite. Although the individual parts of this beekeeping method are not novel in themselves, their combination is innovative. So we will use this term in the following to differentiate between this form of beekeeping and conventional beekeeping practice.

In innovative beekeeping, the drone brood is not removed during mating season (April–June), which drastically increases the mite load in spring and summer. The queen is caged for 25 days within the hive to ensure that the hives are brood free, as the honey bee queen cannot lay eggs during this time. This induces a brood interruption similar to those that occur naturally when colonies swarm in spring and early summer (Seeley and Smith 2015). Caging the queen can reduce *Varroa* mite load by reducing the successful reproduction of the mites (Gabel et al. 2023). The release of the queen when the hives are brood free is followed by an oxalic acid treatment, which typically achieves an efficiency of approximately 90% (Büchler et al. 2020; Charriére and Imdorf 2002). If the mite load is low in winter, no further treatment is required with this method. However, when the recommended threshold values are exceeded, a winter treatment with oxalic acid can be carried out.

In this study, we evaluate *Varroa* mite load and the DWV‐B viral load throughout one season at three different timepoints in honey bee colonies kept according to conventional beekeeping and using the innovative beekeeping method. We took samples from foragers and newly emerged honey bees to determine viral loads. On the one hand, highly infected honey bees can die within a few days after hatching; on the other hand, DWV‐B can replicate throughout the life of a honey bee. In addition, we compared the immune response by counting the total number of haemocytes from 24‐h to 48‐h old honey bees from both treatment groups to determine whether the different *Varroa* levels affect the immune system of honey bees in spring.

## 2 Material and Methods

### 2.1 Honey Bees

The 10 experimental honey bee colonies ( *Apis mellifera carnica* ) were located at the departmental apiary of the University of Würzburg, Germany. All colonies had open‐mated sister queens, which were mated in the same location to minimise genetic variance. The colonies were divided into two comparable groups according to colony strength and *Varroa* mite load in 2021 and have since been continuously managed under the respective treatment. The colonies within each beekeeping practice group were arranged in line next to each other. Colonies from the two beekeeping practices were placed in an L‐shape to minimise the potential drift of bees. We sampled honey bees from four full‐grown colonies (approximately 30,000 worker bees) of each beekeeping practice for our analyses on viruses and immune system functioning to consider differences between honey bees from both beekeeping practices. Conventional management included the removal of drone brood in spring and summer. In April, two drone brood frames were introduced into the colonies, allowing the first drone brood removal to take place around early May. On average, drone brood removal was performed four times, depending on how quickly the colonies rebuilt the brood frames and capped the drone brood. Additionally, formic acid (Formivar, 60%, 174 Andermatt BioVet GmbH, Germany) was vaporised in a Nassenheider Professional evaporator (Joachim Weiland, Werkzeugbau GmbH & Co. KG, Germany) in late summer (detailed information: Table S3) for a summer treatment against *Varroa*. In addition, there was a winter treatment with oxalic acid (Oxuvar, 5.7%, Andermatt BioVet GmbH, Germany). In the colonies maintained under innovative beekeeping practice, the drones developed in the hive and the summer treatment against the *Varroa* mite was performed biotechnically followed by a chemical treatment. The queen was caged in an API‐MO.BRU‐cage (API‐MO.BRU, Mozzato Bruno, Italy) for 25 days until the hive was free of brood. Afterwards, the colonies were treated with oxalic acid (Oxuvar, 5.7%, Andermatt BioVet GmbH, Germany) to reduce the *Varroa* mite load (detailed information: Table S3). There was no treatment at all for the innovatively managed hives in winter. In summary, the main difference between the two methods is that in the innovative beekeeping method, there were fewer treatments against *Varroa* mites compared to the conventional beekeeping method, naturally leading to a higher mite fall in spring and summer. While the management of the colonies according to their respective treatment started in 2021, sample collection for this study was conducted exclusively in 2023 (April–October).

### 2.2 *Varroa* Mite Counting

In order to observe the *Varroa* mite fall of the hives, sticky bottom boards were placed under the hives for 2 days every 2 weeks. The boards were covered with a thin layer of rapeseed oil. This prevents the predation of dead mites by ants and other insects. After these 2 days, the dead mites were counted and the number was divided by two to calculate the number of mites dropped per day.

### 2.3 Haemocyte Counting

The efficiency of the immune system of the honey bees from both beekeeping methods was assessed in early spring. At this time after the winter break in reproduction, the resistance of honey bees determines whether the colony is able to survive and build a strong colony for spring and summer. To investigate the immune system, we caged brood combs from innovatively and conventionally kept hives in an incubator for 1 day. The next day, 20 of the bees that had emerged in the meantime (0–24 h old) from both beekeeping methods were used directly for haemocyte analysis. The remaining emerged bees were transferred to cages to analyse the haemocyte counts the next day (24 h–48 h old). The cages were kept in an incubator (30°C, 50% rh) and the honey bees were supplied with 50% sugar solution and pollen *ad libitum*. Shortly before haemolymph collection, the honey bees were anaesthetized on ice. The honey bees were fixed on a styrofoam plate with crossed needles between thorax and abdomen. A pulled microcapillary (servoprax, A1 0115, servoprax GmbH, Germany) was inserted into the proximal abdomen until 5 μL of haemolymph was collected per honey bee. The haemolymph was mixed directly with 5 μL DAPI staining solution (4′,6‐dia midino‐2‐phenylindole; 50 μg/mL; Invitrogen, Thermo Fisher Scientific, USA). The mixture was immediately transferred to a C‐Chip Neubauer Improved counting chamber (DHC‐N01, Neubauer Improved; NanoEntek Inc. South Korea). Haemocytes were counted under a Zeiss Axiophot fluorescence microscope (Carl Zeiss). This was done with both 0–24 h old and with 24–48 h old honey bees. The experiment was repeated three times with honey bees with a maximum age of 24 h and four times with honey bees with a maximum age of 48 h.

### 2.4 Timepoints and Sampling

The infestation levels of the two groups maintained under different beekeeping methods naturally differ over the course of the year. We therefore took samples of honey bees at three different timepoints during the honey bee season of 2023. We started at the beginning of the season directly after overwintering (April 2023). The second timepoint was just before the *Varroa* treatment in summer (late June 2023). At this time, the *Varroa* mite load differed most strongly, with the mite pressure of innovatively kept colonies being much higher. Another important timepoint was in fall before the colonies went into winter rest (October 2023) (detailed information: Table S3).

We sampled honey bees from eight different hives. Foragers and newly emerged honey bees were asymptomatic for DWV. Five returning honey bee foragers for each colony were sampled using a handheld vacuum cleaner in front of the hive entrance. They were transferred to snap lid jars and immediately immobilised on ice. Newly emerged honey bees were removed directly from the brood combs using snap lid jars and immediately immobilised on ice. The samples were stored at –80°C until analysis.

### 2.5 Processing Samples for qPCR

Only the abdomens were used for RNA extraction, as Yue and Genersch (2005) found DWV in the abdomens of honey bees although they did not show a symptomatic phenotype (Yue and Genersch 2005). The GenUP total RNA kit (biotechrabbit, Henningsdorf, Germany) was used for RNA extraction, following the standard protocol provided by the manufacturer, with the exception that 350 μL instead of 400 μL of the supernatant was transferred to the DNA minifilter. The DNA minifilter was changed to an RNA minifilter and 75% ethanol was added. After several washing steps, 20 μL of RNase‐free water was added to elute the sample until we obtained a total volume of 80 μL. The RNA concentration was measured using the BioPhotometer Plus (Eppendorf SE, Germany) for optical density measurements. The samples were then stored at −80°C until cDNA synthesis.

For cDNA synthesis, we used 400 ng of the extracted total RNA. A cDNA synthesis mix (Biozym cDNA Synthesis Kit, Biozym, Germany) was then prepared according to the manufacturer's protocol. Subsequently, the cDNA was synthesised in a Mastercycler gradient (Eppendorf SE, Germany) using the following program: 42°C (30 min), 85°C (10 min). After synthesis, the cDNA samples were stored at −20°C until analysis.

### 2.6 Standard Curves

To generate standard curves for absolute quantification, we used a PCR product that was DWV‐B positive. We used specific DWV‐B primers for amplification: DWV‐B forward‐primer (5′‐GCCCTGTTCAAGAACATG‐3′) and DWV‐B reverse‐primer (5′‐CTTTTCTAATTCAACTTCACC‐3′) (Locke et al. 2012). To confirm the prevalence of DWV‐B in the PCR product, we used gel electrophoresis and found the expected band at 413 bp. The sample was then purified using the Monarch PCR & DNA Cleanup Kit (New England‐BioLabs, USA). The concentration of the PCR product was measured by optical density and the number of molecules in 1 μL was calculated (1 μg≈2.36 × 10^12^ copies). On this basis, a standard solution was prepared using 8.47 μL of the purified PCR product and the addition of 91.53 μL of DEPC‐H_2_O. Further standard solutions were prepared by doing a 1:10 serial dilution of the previous standard solution (detailed information: Table S1).

Standard curves were generated by analysing standard solutions 3 to 100 (5–50,000,000 copies per reaction) on a Rotor Gene Q (Quiagen, Germany) using the Biozym Blue S'Green qPCR Mix (Biozym, Germany). The resulting standard curve had an efficiency of 96% and was imported into each run in which the DWV viral load was analysed to calculate the absolute quantification of the DWV load in the samples (de Miranda et al. 2013). To calibrate this imported standard curve, the standard solutions four, six, and eight were included in each run to calculate the absolute quantification of the DWV load in the samples.

### 2.7 Realtime qPCR Analyses

For real‐time quantitative PCR analysis (qPCR), a master mix containing Blue S'Green Mix (Biozym, Germany), DEPC‐H_2_O and forward and reverse primers was mixed. The primer sequences and additional details can be found in the Supporting Information (Table S2). We analysed PCR triplicates of each bee's cDNA using the following qPCR protocol: 95°C for 2 min, 40 cycles at 95°C for 5 s and at 60°C for 30 s. The last step consisted of a range from 60°C–90°C with an increase of 1°C for 5 s in a Rotor368 Gene Q (Qiagen, Germany).

### 2.8 Statistics

Statistical analyses were performed with R version 4.1.2 including “stats” (R Core Team 2021). Additionally, the package “rstatix‐version 7.0.7” (Kassambara 2021) was used for further statistical analyses. To implement the data, the following packages were used: “readxl‐version 1.3.1” (Wickham and Bryan 2019), “dplyr‐version 1.0.7” (Wickham et al. 2021) and “tidyverse‐version 1.3.1” (Wickham et al. 2019). Graphs were created with the packages “ggplot2‐version 3.4.4” (Wickham 2016) and “ggpubr‐version 0.4.0” (Kassambara 2020). As the data were not normally distributed (Shapiro–Wilk normality test), a Wilcoxon rank sum test was performed to compare groups. A Kruskal–Wallis test was used to analyse viral quantities, followed by multiple comparison Dunn's test with Bonferroni correction. Correlation data were analysed using a linear correlation and a correlation with a Spearman's rank correlation.

## 3 Results

### 3.1 Number of Fallen *Varroa* Mites

At the beginning of the honey bee season in April, innovatively kept colonies had already significantly higher levels in mite fall compared to conventionally kept colonies (Table 1; *W* = 0.5, *p* = 0.036, Wilcoxon rank sum test). At the second timepoint in June, the difference between the two beekeeping methods was even more drastic, and the innovative group had about 30 times higher numbers of fallen *Varroa* mites (*W* = 0, *p* = 0.028, Wilcoxon rank sum test). After summer treatment and shortly before overwintering, i.e., in October, both groups had a similar number in mite fall (*W* = 8, *p* = 1, Wilcoxon rank sum test).

**TABLE 1:** *Varroa* mite fall at three selected timepoints. Numbers of naturally fallen mites per day using sticky bottom boards. Hive 1, 2, 3, and 5 were kept according to innovative beekeeping; hive 6, hive 6, 8, 9 and 10 were maintained conventionally.

Hive	Treatment	First timepoint 11 April 2023	Second timepoint 29 June 2023	Third timepoint 11 October 2023
1	Innovative	1	85	4
2	Innovative	4	54	1
3	Innovative	15	43	10
5	Innovative	4	57	6
6	Conventional	1	3	3
8	Conventional	0	1	4
9	Conventional	0	3	4
10	Conventional	0	1	11

### 3.2 Haemocytes

**FIGURE 1:**
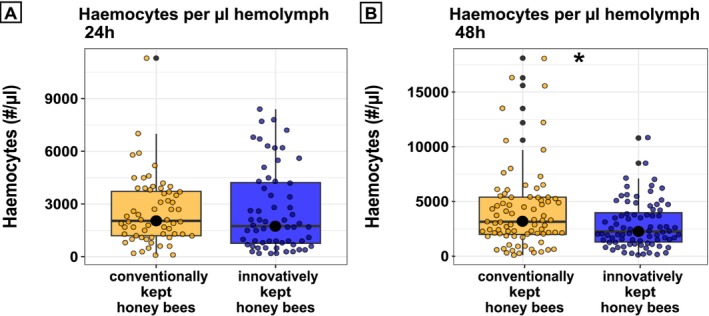
Total haemocyte counts in 0–24 h old (A) and 24–48 h old honey bees (B). Bees from both beekeeping methods did not differ in their total haemocyte counts when younger than 24 h (*n* = 60 honey bees per treatment) (A). Honey bees (conventional vs. innovative beekeeping) between 24 h and 48 h after emerging differed significantly in their haemocyte counts (*n* = 80 honey bees per treatment). Boxplots showing the distribution of haemocytes. The median is represented by the central line with a black filled dot in the middle, while the boxes indicate the interquartile range (IQR). Whiskers represent the data range, and black points outside the whiskers are considered outliers. Jitter points represent the mean number of haemocytes per μL haemolymph of an individual honey bee. Significant differences are indicated by asterisks *: *p* < 0.05. As the data were not normally distributed, a Wilcoxon rank‐sum test was used to assess statistical differences between treatment groups.

In honey bees younger than 24 h, no differences in haemocyte counts were found (Figure 1A, *W* = 1933, *p* = 0.487; Wilcoxon rank sum test). However, haemocyte counts differed significantly 48 h after emerging of the bees between both groups (Figure 1B, *W* = 3865.5, *p* = 0.023, Wilcoxon rank sum test).

### 3.3 Correlation of *Varroa* Mite Fall and DWV‐B Viral Load

**FIGURE 2:**
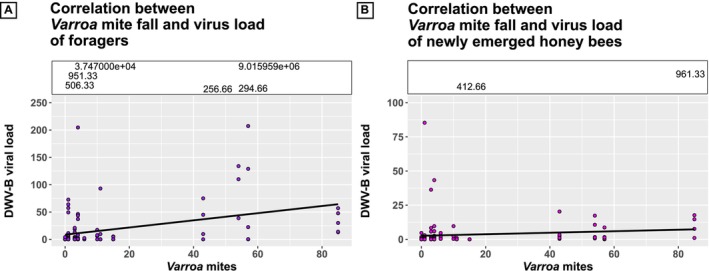
Correlation of *Varroa* mite fall and DWV‐B viral load in honey bees in foragers (A) and newly emerged honey bees (B). In foragers (purple data points) and newly emerged honey bees (pink data points), DWV‐B viral load correlated positively with *Varroa* mite fall (foragers: *ρ* = 0.306, *p* = 0.001; newly emerged honey bees: *ρ* = 0.246, *p* = 0.007). The relationship between the number of fallen mites and DWV‐B viral loads were fitted to a linear regression function. Outliers were included in the statistical analysis but are shown numerically in a box at the top of the graph to improve visualisation.

The viral load of DWV‐B was higher in honey bee colonies with a high *Varroa* mite fall, that is, colonies maintained according to innovative beekeeping. This phenomenon was observed in foragers (Figure 2A, *ρ* = 0.306, *p* = 0.001, Spearman's rank correlation) and in newly emerged honey bees (Figure 2B, *ρ* = 0.246, *p* = 0.007, Spearman's rank correlation).

### 3.4 Course of DWV‐B Viral Loads Over the Season

**FIGURE 3:**
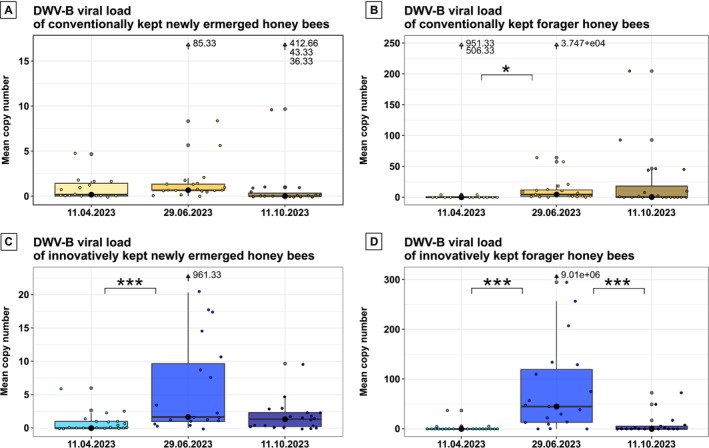
DWV‐B viral loads over the course of the season. DWV‐B viral load of newly emerged honey bees (A and C) and foragers (B and D) of both beekeeping method groups during the bee season. The viral load of newly emerged honey bees stayed low and did not differ between timepoints in bees maintained according to conventional beekeeping (A). Foragers of conventionally kept honey bees differed in their viral loads from the first timepoint in April onwards. There was a significant increase in DWV‐B virus between April and June (B). The viral load of newly emerged honey bees of the innovatively kept honey bees differed between the timepoints. From April to June, viral loads increased significantly (C). Foragers of the innovatively kept hives also showed differences between timepoints. An increase of DWV‐B viral load from April to June and a significant decrease from June to October were found (D). Boxplots showing the distribution of DWV‐B viral loads. The median is represented by the central line with a black filled dot in the middle, while the boxes indicate the interquartile range (IQR). Whiskers represent the data range, and black points outside the whiskers are considered outliers. Jitter points represent the mean DWV‐B copy number for individual honey bees. Significant differences between timepoints are indicated by asterisks: *: *p* < 0.05; **: *p* < 0.01; ***: *p* < 0.001. Outliers were included in the statistical analysis but are shown numerically at the top of the graph to improve visualisation. As the data were not normally distributed, a Kruskal–Wallis test was used to assess statistical differences between timepoints, followed by Dunn's test for multiple comparisons.

Conventional beekeeping aims to constantly minimise the level of infestation with several treatments against *Varroa*. As expected, the DWV‐B viral load of conventionally kept hives remained low throughout the season. There were no differences in the viral load in newly emerged honey bees of conventionally kept honey bees between timepoints (Figure 3A; *χ* ^2^ = 4.220, df = 2, *p*‐value = 0.121; Kruskal‐Wallis). Foragers differed significantly in their viral loads throughout the season (Figure 3B; *χ* ^2^ = 8.163, df = 2, *p*‐value = 0.017, Kruskal–Wallis). Conventionally kept foragers had significantly lower viral loads in April compared to the end of June (Dunn's test, *p*‐value = 0.016).

The innovatively kept colonies were confronted with a high mite load during the season (Table 1). This affected the viral load and resulted in viral load differences between timepoints found in newly emerged honey bees (*χ* ^2^ = 14.535, df = 2, *p*‐value < 0.001, Kruskal–Wallis, Figure 3C,D). Even though the DWV‐B viral load was low in April, there was a drastic increase in viral load during the summer (Dunn's test, *p*‐value < 0.001). In foragers maintained according to innovative beekeeping, timepoint similarly affected viral load (*χ* ^2^ = 28.305, df = 2, *p*‐value < 0.001, Kruskal–Wallis). Viral load was significantly higher at the end of June compared to April (Dunn's test, *p*‐value < 0.001). In addition, a significant decrease in viral load occurred from June to October (Dunn's test, *p*‐value < 0.001).

### 3.5 Differences in DWV‐B Viral Loads due to *Varroa* Infestation at Three Crucial Timepoints

**FIGURE 4:**
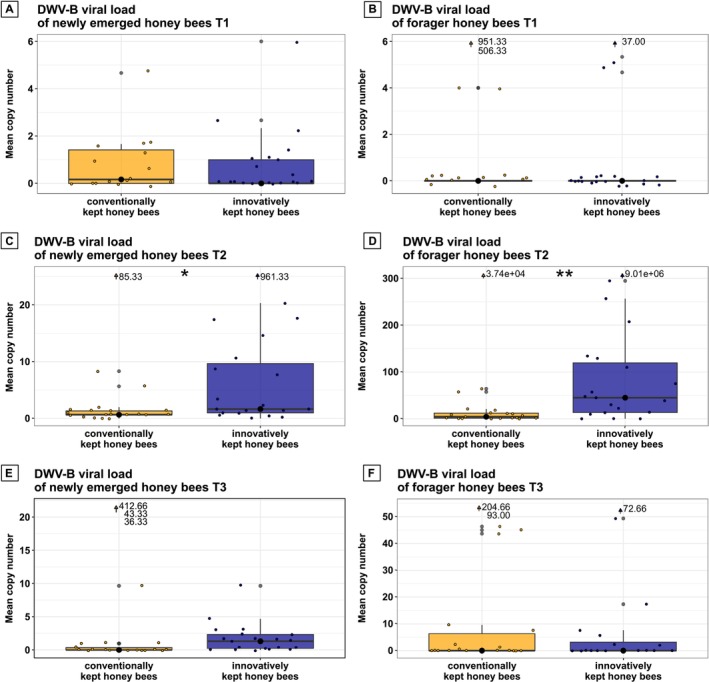
Differences in DWV‐B viral load between bees of both beekeeping methods. DWV‐B viral load of newly emerged honey bees (A) and foragers (B) of conventionally versus innovatively kept honey bees in April. The viral load of newly emerged honey bees did not differ between groups. Foragers of both beekeeping methods also did not differ in their DWV‐B loads. At the end of June, viral loads of newly emerged honey bees (C) and foragers (D) of innovatively kept honey bees were higher compared to those of the conventionally kept bees. Shortly before overwintering in October, DWV‐B viral loads of newly emerged honey bees (E) and foragers (F) did not differ between bees of both beekeeping methods. Values of the outliers were shown numerically at the top of the graph. Boxplots showing the distribution of DWV‐B viral loads. The median is represented by the central line with a black filled dot in the middle, while the boxes indicate the interquartile range (IQR). Whiskers represent the data range, and black points outside the whiskers are considered outliers. Jitter points represent the mean DWV‐B copy number for individual honey bees. Significant differences between timepoints are indicated by asterisks: *: *p* < 0.05; **: *p* < 0.01, ***: *p* < 0.001. Outliers were included in the statistical analysis but are shown numerically at the top of the graph to improve visualisation. As the data were not normally distributed, a Wilcoxon rank‐sum test was used to assess statistical differences between treatment groups.

In April, when honey bee workers and *Varroa* mites increased in numbers, there were no differences in DWV‐B viral load between bees from both beekeeping methods in newly emerged honey bees (*W* = 169, *p* = 0.769; Wilcoxon rank‐sum test) and in foragers (*W* = 167.5, *p* = 0.417, Wilcoxon rank‐sum test). The DWV‐B viral load was comparatively low in foragers (Figure 4A,B).

*Varroa* mite load increased steadily with the advancing season. Especially for the innovatively kept colonies, the infestation rate was growing fast. Here, the drone brood was not removed, which likely led to the increase in viral load (Figure 4C,D). Shortly before the summer treatment against the *Varroa* mite, the viral load in the innovative beekeeping group was significantly higher in newly emerged honey bees (*W* = 117, *p* = 0.025, Wilcoxon rank‐sum test). The viral load of the foragers from the innovatively kept hives was also significantly higher than that of the foragers from conventionally kept hives (*W* = 99, *p* = 0.006, Wilcoxon rank‐sum test).

Viral load was again low in newly emerged bees of both treatment groups in October after all hives had undergone the summer treatment (Figure 4E, *W* = 143.5, *p* = 0.114, Wilcoxon rank‐sum test). The same phenomenon was apparent in foragers of both beekeeping methods (*W* = 188, *p* = 0.561, Wilcoxon rank‐sum test). No significant differences in viral loads were found between foragers from both beekeeping groups in October (see Figure 4F).

## 4 Discussion

### 4.1 Seasonal Dynamics of Mite Load and DWV‐B Viral Load

**FIGURE 5:**
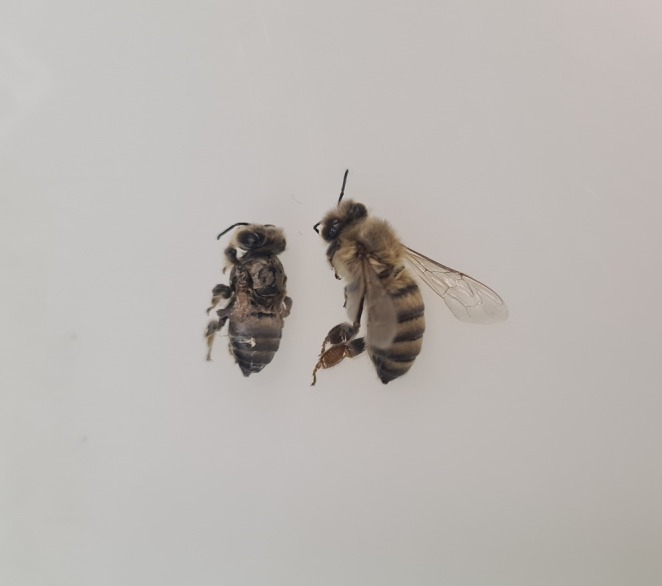
Newly emerged honey bee with symptoms of DWV infection (left) and newly emerged honey bee without symptoms (right). Compared to the asymptomatic honey bee, the DWV infected bee showed the classic DWV symptoms, that is, crippled wings, discoloration, and a shortened abdomen.

The *Varroa* mite is the dominant factor threatening honey bee health and leading to losses of entire honey bee colonies. The mite weakens the bees by consuming their fat body and haemolymph and by transferring pathogens such as DWV. The virus leads to crippled bees unable to forage (Figure 5). Frequent treatment against the mite can reduce the mite load but inhibits adaptation of the honey bee host to the mite and might induce resistance in the mites (Rinkevich 2020). We here compared two beekeeping methods differing in the *Varroa* mite treatment and therefore in the *Varroa* load experienced by the bees. Our aim was to understand to how far a high seasonal mite load would affect honey bee health, while allowing the bees the opportunity to adapt to their parasite.

The seasonal dynamics of *Varroa* mite fall correlated significantly with DWV‐B viral loads. Similar correlation values were reported by Molinatto et al. (2025) in Italian and French honey bee colonies. Innovatively kept hives were exposed to a higher *Varroa* mite pressure in spring and summer than conventionally kept hives, mainly because drone brood had not been removed. *Varroa* mites prefer drone brood over worker brood because of the longer developmental time of the drones (Currie 1987; Fuchs 1990). Generally, newly emerged honey bee hosts showed a lower viral load than foragers at all timepoints and in both beekeeping groups (Figures 3 and 4). This is in line with data from Ongus et al. (2004) showing that DWV‐B replicates in honey bees over time. Low virus titers likely increase with adult maturation within the individual honey bee host. Additionally, honey bees can also be infested for the first time during nursing tasks since *Varroa* mites favour nurse bees as hosts for the dispersal phase (Xie et al. 2016). Due to cleaning activity and foraging on contaminated flowers, there is also a high risk for further infection with DWV‐B (Deutsch et al. 2023; de Miranda et al. 2013). DWV‐B viral load of the innovatively kept honey bees increased from April to June in foragers and newly emerged honey bees. The high viral load observed in summer in foragers was strongly reduced again in the fall measurement, likely as an indirect effect of the innovative summer treatment, which effectively reduced the parasitic mite infestation. However, bees maintained under innovative beekeeping usually had higher viral loads compared to the conventionally kept honey bees. Whether the higher mite pressure leads to long‐term effects on the colony needs further investigation.

Foragers from conventionally maintained colonies had significantly increasing DWV‐B viral loads from April to June, in line with the literature (Glenny et al. 2017; Locke et al. 2017; Traynor et al. 2016; Martin et al. 2010). Our data substantiate the positive correlation between *Varroa* mite fall and DWV‐B viral load (Francis et al. 2013; Barroso‐Arévalo et al. 2019).

Our data further imply that despite the high *Varroa* load in summer, innovative beekeeping can effectively reduce the number of mites and pathogens by the summer treatment consisting of queen caging and oxalic acid treatment. This approach may provide the opportunity for honey bees to adapt to the parasitic mite in spring and summer while ensuring the production of healthy and almost mite‐free winter bees after the summer treatment to secure a healthy overwintering period.

Importantly, our data indicate that the colonies in the innovative beekeeping concept were not only healthy in fall but also had sufficient winter stores, as the amount of honey yielded in spring and summer did not differ between the two groups (Data not shown). This indicates that neither the summer treatment with induced brood interruption nor the increased virus and mite load in spring and summer significantly affected resource accumulation. This is in line with the findings of Kovačić et al. (2023), who showed that summer brood interruption followed by oxalic acid treatment has no negative effects on colony strength or honey yield, provided it is carried out at the right time. However, further studies with a larger number of honey bee colonies are required in order to obtain more reliable conclusions regarding colony development and honey production.

DWV‐B is not only transmitted vertically by queens and drones and by *Varroa* infestation, but also via larval food produced in the hypopharyngeal glands of honey bees (Yue and Genersch 2005; Yue et al. 2007). Therefore, a brood interruption in summer, which mimics the natural brood interruption caused by swarming, typically occurring in spring and early summer, is highly effective in reducing pathogens in nurse bees and consequently in larvae raised after the interruption. Similarly, a winter brood interruption induced by cold temperatures is an important natural way of reducing mites and pathogens.

### 4.2 Mite Load Affects Immune Response

An impaired immune response can severely affect lifespan, disease susceptibility, and survival of the honey bee colony (Negri et al. 2016). To test whether *Varroa* infestation affects immune system functioning (Le Conte et al. 2010; Yang and Cox‐Foster 2005; Koleoglu et al. 2018), we compared the haemolymph haemocytes of newly emerged honey bees from both beekeeping groups. In spring, the increased *Varroa* mite fall observed in the innovatively kept bees was associated with a reduced number of haemocytes in bees from innovative beekeeping compared to bees from conventional beekeeping (Figure 1B). This supports the notion that the basal cellular immunocompetence is impaired by the elevated *Varroa* infestation (Koleoglu et al. 2018). Nevertheless, future studies should evaluate other aspects of the immune system, such as phenoloxidase (PO) activity and the expression of antimicrobial peptides (AMPs), to provide a better understanding of how *Varroa* pressure affects the immune system of honey bees (Laughton et al. 2011). One explanation for the reduced number of haemocytes in the haemolymph of innovatively kept bees is that proteins in the saliva of *Varroa* mites might reduce the total haemocyte counts in honey bees (Richards et al. 2011). Haemocytes are necessary to recognise and respond to foreign surfaces in the haemocoel (Marmaras and Lampropoulou 2009; Negri et al. 2016) and have an important function in wound closure (Negri et al. 2016). The mite's feeding hole serves both as a food source and as an entry for pathogens. *Varroa* infestation alters the physiology, which normally changes with the age of honey bees (Amdam et al. 2004). Usually, older honey bees have higher levels of juvenile hormone (Bloch et al. 2002). Zanni et al. (2018) showed that seven‐day‐old honey bees infested with *Varroa* mites had higher levels of juvenile hormone compared to uninfected honey bees. Juvenile hormone is further associated with a decrease in haemocytes (Amdam and Aase 2005). We observed lower haemocyte levels after 48 h in honey bees, but we could not detect any differences between both beekeeping methods 24 h after emergence. A possible explanation for this could be the prolonged contact time between individuals in the cages, which may have increased their exposure to pathogens from other bees that had already been infested with *Varroa* during development. Additionally, foodborne transmission of pathogens could have contributed to the observed differences. It is also possible that mites emerged with their hosts switching to other individuals after emerging, further influencing their immune response.

## 5 Conclusion

Our results demonstrate that an induced brood interruption in summer followed by oxalic acid treatment is an option to support the opportunity of the honey bee host to adapt to its major parasite, the *Varroa* mite, without being killed by the mite or viruses transmitted by the parasitic mite. Further studies need to investigate whether honey bees from the innovative beekeeping method also exhibit behavioral changes, such as *Varroa* sensitive hygiene and recapping behavior, to show that confrontation with *Varroa* in spring and summer promotes resistance traits and leads to adaptation in behavior towards *Varroa* resistance. Mite fall correlated with viral load and challenged the immune system of the bees. Despite the high *Varroa* pressure in spring and summer, winter bees emerging after this summer treatment were strong and healthy, meaning that the threat of winter losses is not greater than in hives maintained conventionally. It is important to note that this study was conducted in a temperate climate zone. Environmental factors such as climate conditions and forage abilities may influence the effectiveness of this method. An additional advantage of innovative beekeeping is not only that the bees have the opportunity to adapt to the *Varroa* mite over time but also that summer treatment is more insensitive to external factors for example, the temperature. The innovative beekeeping method is therefore better suited to the challenges posed by climate change compared to conventional treatments with formic acid, which is highly temperature dependent and is typically evaporated over a period of 2 weeks. During our experiments in 2023, extreme weather conditions underlined these restrictions. In July, temperatures reached 38.8°C, and September had several days above 30°C (DWD 2023). Such conditions make the use of formic acid treatments particularly risky. Therefore, finding alternative mite control strategies is indispensable.

## Author Contributions

**Lioba Hilsmann:** writing – original draft, investigation, methodology, validation, visualization, writing – review and editing. **Lena Wolf:** writing – review and editing, methodology, validation, investigation. **Markus Thamm:** writing – review and editing, methodology. **Sylvie Vandenabeele:** writing – review and editing, methodology. **Ricarda Scheiner:** writing – original draft, supervision, writing – review and editing, funding acquisition, conceptualization.

## Disclosure

All claims expressed in this article are solely those of the authors and do not necessarily represent those of their affiliated organisations, or those of the publisher, the editors and the reviewers. Any product that may be evaluated in this article, or claim that may be made by its manufacturer, is not guaranteed or endorsed by the publisher.

## Ethics Statement

Our protocols comply with standard welfare practice in our field.

## Conflicts of Interest

The authors declare no conflicts of interest.

## Supporting information


**TABLE S1.** Standard solutions to create standard curves for absolute quantification. Standard solutions of a positive PCR product for DWV‑B were created to establish standard curves for absolute quantification.
**TABLE S2.** Primers used for the gene expression analysis. The respective gene, the sequence of the corresponding forward and reverse primer and the expected length of the product are shown. *Deformed wing virus B* primers were ordered as published before (Locke et al. 2012).
**TABLE S3.** *Varroa* mite fall over the sampling period with dates of the respective treatments. Treatments of the innovative approach are bordered in blue, whereas those of the conventional method bordered in yellow. Sample timepoints highlighted in orange.

## Data Availability

The data that support the findings of this study are available on request from the corresponding author. The data are not publicly available due to privacy or ethical restrictions.
