# Supplementary material for: Towards a Stable Host–Parasite Relationship Between Honey Bees and Varroa Mites Through Innovative Beekeeping
Source: Environ Microbiol. 2025 May 1;27(5):e70101. doi: 10.1111/1462-2920.70101 (PMC12045652; doi:10.1111/1462-2920.70101)
Supplement: Supplementary file 1 — TABLE S1. Standard solutions to create standard curves for absolute quantification. Standard solutions of a positive PCR product for DWV‑B were created to establish standard curves for absolute quantification. TABLE S2. Primers used for the gene expression analysis. The respective gene, the sequence of the corresponding forward and reverse primer and the expected length of the product are shown. Deformed wing virus B primers were ordered as published before (Locke et al. 2012). TABLE S3. Varroa mite fall over the sampling period with dates of the respective treatments. Treatments of the innovative approach are bordered in blue, whereas those of the conventional method bordered in yellow. Sample timepoints highlighted in orange. [file EMI-27-e70101-s001.docx]

**Table S1: Standard solutions to create standard curves for absolute quantification.** Standard solutions of a positive PCR product for DWV‑B were created to establish standard curves for absolute quantification.

| Standard solution | Copy numbers per run |
| --- | --- |
| 3 | 50.000.000 |
| 4 | 5.000.000 |
| 5 | 500.000 |
| 6 | 50.000 |
| 7 | 5.000 |
| 8 | 500 |
| 9 | 50 |
| 10 | 5 |

**Table S2: Primers used for the gene expression analysis.** The respective gene, the sequence of the corresponding forward and reverse primer and the expected length of the product are shown. *Deformed wing virus B* primers were ordered as published before (Locke et al., 2012).

| Gene | Primer | Sequence | Length  [bp] | T_m_ | Reference |
| --- | --- | --- | --- | --- | --- |
| *Deformed wing virus B* | forward  reverse | 5′-GCCCTGTTCAAGAACATG -3′  5′- CTTTTCTAATTCAACTTCACC-3′ | 413 | 80.5 °C | Locke et al., 2012 |

**Table S3: *Varroa* mite fall over the sampling period with dates of the respective treatments.** Treatments of the innovative approach are bordered in blue, whereas those of the conventional method bordered in yellow. Sample timepoints highlighted in orange.

| Date | Hive 1 | Hive 2 | Hive 3 | Hive 5 | Hive 6 | Hive 8 | Hive 9 | Hive 10 |
| --- | --- | --- | --- | --- | --- | --- | --- | --- |
| 02.03.2023 | 1 | 1 | 0 | 1 | 0 | 1 | 0 | 1 |
| 16.03.2023 | 1 | 0 | 4 | 1 | 1 | 0 | 1 | 1 |
| 30.03.2023 | 1 | 2 | 9 | 9 | 2 | 0 | 0 | 1 |
| 11.04.2023 | First sample timepoint | | | | | | | |
| 13.04.2023 | 1 | 4 | 15 | 4 | 1 | 0 | 0 | 0 |
| 27.04.2023 | 11 | 8 | 34 | 15 | 2 | 0 | 1 | 0 |
| 11.05.2023 | 15 | 8 | 19 | 31 | 1 | 0 | 1 | 0 |
| 25.05.2023 | 14 | 14 | 31 | 43 | 1 | 2 | 1 | 0 |
| 08.06.2023 | 38 | 40 | 44 | 63 | 3 | 1 | 2 | 1 |
| 22.06.2023 | 60 | 49 | 32 | 63 | 0 | 0 | 2 | 2 |
| 29.06.2023 | Second sample timepoint | | | | | | | |
| 30.06.2023 | Queen caging | | | |  | | | |
| 06.07.2023 | 85 | 54 | 43 | 57 | 3 | 1 | 3 | 1 |
| 20.07.2023 | 115 | 89 | 111 | 106 | 2 | 3 | 2 | 2 |
| 25.07.2023 | Treatment with oxalic acid | | | |  | | | |
| 28.07.2023 | 809 | 433 | 407 | 560 | 0 | 1 | 1 | 3 |
| 31.07.2023 | 133 | 56 | 60 | 64 | 0 | 3 | 3 | 3 |
| 02.08.2023 | 13 | 6 | 4 | 9 | 0 | 3 | 1 | 8 |
| 04.08.2023 | 7 | 5 | 2 | 4 | 1 | 2 | 3 | 7 |
| 07.08.2023 | 5 | 2 | 1 | 2 | 5 | 4 | 3 | 7 |
| 10.08.2023 | 0 | 0 | 0 | 0 | 1 | 2 | 3 | 2 |
| 10.08.2023 |  | | | | Treatment with formic acid | | | |
| 14.08.2023 | 1 | 2 | 1 | 1 | 41 | 35 | 77 | 91 |
| 16.08.2023 | 1 | 2 | 2 | 1 | 3 | 3 | 27 | 20 |
| 18.08.2023 | 3 | 0 | 1 | 2 | 7 | 21 | 9 | 26 |
| 21.08.2023 | 1 | 1 | 3 | 1 | 10 | 17 | 4 | 17 |
| 23.08.2023 | 2 | 1 | 2 | 1 | 6 | 15 | 5 | 16 |
| 25.08.2023 | 3 | 1 | 1 | 2 | 2 | 13 | 5 | 5 |
| 28.08.2023 | 0 | 0 | 1 | 1 | 0 | 2 | 1 | 0 |
| 30.08.2023 | 1 | 1 | 1 | 2 | 0 | 0 | 1 | 0 |
| 14.09.2023 | 5 | 0 | 5 | 3 | 1 | 0 | 1 | 2 |
| 28.09.2023 | 12 | 1 | 8 | 2 | 0 | 5 | 2 | 4 |
| 11.10.2023 | Third sample timepoint | | | | | | | |
| 12.10.2023 | 4 | 1 | 10 | 6 | 3 | 4 | 4 | 11 |
| 27.10.2023 | 11 | 0 | 2 | 7 | 2 | 3 | 3 | 13 |
